# The Benefits of Higher Income in Protecting against Chronic Medical Conditions Are Smaller for African Americans than Whites

**DOI:** 10.3390/healthcare6010002

**Published:** 2018-01-09

**Authors:** Shervin Assari

**Affiliations:** 1 Center for Research on Ethnicity, Culture, and Health (CRECH), School of Public Health, University of Michigan, Ann Arbor, MI 48104, USA; assari@umich.edu; Tel.: +1-734-363-2678; 2 Department of Psychiatry, University of Michigan, 4250 Plymouth Rd., Ann Arbor, MI 48109-2700, USA

**Keywords:** social and economic inequalities, racial and ethnic health disparities, socioeconomic status, chronic disease

## Abstract

**Background:** Blacks’ diminished return is defined as smaller protective effects of socioeconomic status (SES) on health of African Americans compared to Whites. Aim: Using a nationally representative sample, the current study aimed to examine if the protective effect of income on chronic medical conditions (CMC) differs for African Americans compared to Whites. **Methods:** With a cross-sectional design, the National Survey of American Life (NSAL), 2003, included 3570 non-Hispanic African Americans and 891 non-Hispanic Whites. The dependent variable was CMC, treated as a continuous measure. The independent variable was income. Race was the focal moderator. Age, education, and marital status were covariates. Linear regressions were used to test if the protective effect of income against CMC varies by race. **Results:** High income was associated with a lower number of CMC in the pooled sample. We found a significant interaction between race and income, suggesting that income has a smaller protective effect against CMC for African Americans than it does for Whites. **Conclusion:** Blacks’ diminished return also holds for the effects of income on CMC. Blacks’ diminished return is a contributing mechanism to the racial disparities in health in the United States that is often overlooked. More research is needed on the role of diminished health return of SES resources among other minority groups.

## 1. Introduction

With few exceptions, all state-of-the-art longitudinal studies that have been conducted across the globe have documented the protective effects of socioeconomic status (SES) indicators on population health [1,2,3,4,5,6]. SES indicators, such as income, protect individuals against a wide range of health problems including but not limited to morbidity [7] and mortality [8,9,10]. Although other SES indicators such as education [11] and employment [3,12,13] are also important, income is one of the most salient SES indicators [14].

Population sub-groups, however, differ in the protective effects of SES on health. Age [15], gender [3,15,16,17,18,19], neighborhood SES [20,21,22], race [23,24,25] and their intersections [23] may alter the link between SES and health. According to the “Blacks’ diminished return” hypothesis, the protective effects of SES indicators are systemically smaller for African Americans than Whites [18,26,27]. Similar effects are reported for the effects of education [24] and employment [28] on mortality and mental health [23]. However, it is unknown whether the same pattern holds for the effects of SES indicators, such as income, on chronic medical conditions (CMC).

Still, we do not know if it is race and SES or race or SES that impact health. If it is race or SES, SES will fully explain the effects of race on health [28,29,30]. If the protective effects of SES indicators vary across races [31,32], it is race and SES together that offer protection from health problems. Previous research has suggested that the “race and place” hypothesis may be more plausible that the “race or place” hypothesis [26,27]. In the United States (U.S.), residential segregation results in different access to the opportunity structure for Whites and Blacks [33]. Despite similar income, wealth and purchasing power are lower among African Americans than Whites [34]. As a result, education, employment, and income may have a larger influence in changing life conditions for Whites compared to African Americans [35,36]. It is, however, still unknown whether the effects of income against CMC also depend on race or not.

### Aims

Using a nationally representative sample of African American and White adults in the U.S. [37], the current study tested whether the protective effect of income on the number of CMC varies dependent on race.

## 2. Methods

With a cross-sectional design, the current study used data from the National Survey of American Life (NSAL), 2003. The NSAL was conducted by the University of Michigan (UM) as a part of the Collaborative Psychiatric Epidemiology Surveys (CPES). Although the NSAL methodology has been described elsewhere [37], we briefly describe the study here.

### 2.1. Ethical Considerations

The study was approved by the Institutional Review Board (IRB B03-00004038-R1) at the University of Michigan (UM). Written consent was received from all participants. Data were restored and analyzed anonymously. All participants were financially compensated for their time.

### 2.2. Sampling and Participants

The NSAL is a household survey of Blacks with a White control group. The study used a national household probability sampling to recruit White and Black American adults (18 years of age and older). African Americans and Whites in the NSAL were selected from large cities, other urban areas, or rural areas. All Blacks and Whites in the NSAL are non-Hispanic.

Concerning race, Blacks or African-Americans in this study indicate self-identified Blacks without any ancestral ties to the Caribbean [38].

### 2.3. Enrollment in the NSAL

The NSAL sample was composed of African American, Caribbean Black, or White American adults (aged 18 years and older). All of the participants were sampled from households located in the coterminous 48 states. The NSAL sample was restricted to individuals who could conduct a structured interview in English. Institutionalized individuals were not enrolled in the study. Individuals who were in prisons, jails, nursing homes, and long-term medical or dependent care settings were excluded [38]. The current study only used data of African Americans and Whites, but not Caribbean Blacks.

### 2.4. Sampling

The NSAL used a multi-stage sampling design to enroll participants. The NSAL used a core and a supplemental sample. The NSAL core sample was a national probability sample for the sampling of African Americans and Whites. The NSAL core sample was similar to the National Survey of Black Americans (NSBA), 1979–1980 sampling, which is believed to be optimal for drawing a Black sample. The NSAL supplemental sample was composed of households in areas of high Caribbean Black residential density. This study focused on a comparison of African Americans and Whites, thus we did not include the Caribbean Black sample in this paper [38].

### 2.5. Data Collection

NSAL used structured interviews using survey questionnaires for data collection. Eighty-six percent of the interviews were face-to-face computer-assisted interviews. Telephone interviews were used for the remaining 14% of data collection. Interviews lasted 2 h and 20 min on average. Response rate was 70.7% for African Americans, and 69.7% for Whites.

### 2.6. Measures

#### 2.6.1. Moderator

Race and ethnicity were self-identified in the current study. All of the participants were either non-Hispanic White or non-Hispanic African American. Race was treated as a categorical variable in the current study (African Americans = 1 versus Whites = 0 (reference category)).

#### 2.6.2. Covariates

Demographic characteristics in the current study included age and gender. Age was conceptualized as a continuous measure, and gender was a dichotomous variable (with male as the reference group). Socioeconomic covariates included education and marital status. Education was operationalized as a dichotomous variable (≥12 years versus 11 years or less (reference category)) which reflected the completion of secondary school [39]. Similar to previous literature [40], marital status was also treated as a dichotomous variable (married = 1 versus non-married = 0 (reference category)).

#### 2.6.3. Independent Variable

Household income was the primary independent variable in this study. Household income was self-reported and top coded at USD200,000 (35 individuals reported income more than USD200,000). Income was treated as a continuous measure in this study. To make regression coefficients more interpretable, we divided income by USD10,000. So, our regression coefficients reflect the effect of each USD10,000 change in income on CMC. 

#### 2.6.4. Dependent Variable

CMCs were measured using self-reported history of doctor-diagnosed chronic medical conditions, from the following 14 medical conditions: diabetes, arthritis/rheumatism, peptic ulcers, cancer, hypertension, chronic liver disease, chronic kidney disease, stroke, asthma, other chronic lung diseases, atherosclerosis, sickle cell disease, heart disease, and glaucoma. To measure CMC, participants were asked whether a doctor has ever told them that they have the above listed conditions. We calculated a CMC score, which reflected number of CMC with a potential range from 0 to 14, with a higher score indicating more CMC. CMC was treated as a continuous measure in this study. Self-reported measures of CMC have acceptable validity and reliability [41].

### 2.7. Statistical Analysis

To accommodate the NSAL’s multi-stage sample design, and to apply the NSAL weights due to strata, clusters, and non-response, we used Stata 13.0 (Stata Corp., College Station, TX, USA) to perform data analysis. Taylor series linearization was applied for the estimation of standard errors. To use a subsample analysis framework, we used sub-population survey commands for all our analyses. For descriptive statistics, we used mean and proportions (frequencies). For bivariate correlations, we used independent sample *t* test, Pearson Chi square, and Pearson correlation tests. From Pearson correlation tests, we reported *r* values. For multivariable analysis, we used a number of linear regression models. 

In these models, we used income as the independent variable, the number of comorbid medical conditions as the dependent variable, socio-demographics as controls, and race as the focal moderator. First, two linear regressions were estimated in the pooled sample of African Americans and Whites. The first model did not include race by income interaction. We then ran a model with race by income interaction term. Subsequently, we performed race/ethnic-specific linear regressions (Model 3 for Whites and Model 4 for Blacks). Adjusted Beta (B) and 95% Confidence Intervals (CI) were reported. *p*-values less than 0.05 were considered significant.

## 3. Results

### 3.1. Descriptive Statistics

Table 1 provides descriptive statistics for the pooled sample as well as for Whites and African Americans. Compared to Whites, African Americans had lower educational attainment and income. Also, fewer African Americans were married in comparison to Whites (Table 1).

### 3.2. Bivariate Correlations

Table 2 presents the results of bivariate correlations for the pooled sample as well as separately for Whites and African Americans. Income showed a moderate and negative association with the number of CMC in the pooled sample (*r* = −0.15), Whites (*r* = −0.21), and African Americans (*r* = −0.14). Age showed a modest and positive correlation with the number of CMC in the pooled sample (*r* = 0.48), Whites (*r* = 0.47), and African Americans (*r* = 0.49) (Table 2).

### 3.3. Linear Regressions in the Pooled Sample

Table 3 presents the results of two linear regression models in the pooled sample with income as the independent variable and the number of CMC as the dependent variable. Model 1 only included the main effects. Model 2 also included an interaction term between race and income. Based on Model 1, high income had a protective effect against the number of CMC above and beyond all of the covariates. Model 2 showed a significant interaction between the effects of race and income on the number of CMC, suggesting that the protective effect of income against the number of CMC is smaller for African Americans than it is for Whites (Table 3).

### 3.4. Linear Regressions in Whites and African Americans

Table 4 summarizes the results of two linear regression models that were estimated separately for Whites and African Americans. Models 3 and 4 show that in Whites and Blacks, high income was associated with a lower number of CMC; however, the magnitude of the association was stronger for Whites than it is for African Americans (regression coefficient of −0.07 versus −0.04 for Whites compared to African Americans; Table 4).

## 4. Discussion

Based on our findings, an increase in income is associated with a disproportionately larger decline in the number of CMC for Whites compared to African Americans. That is, although Whites and African Americans both gain physical health as their income increases, this protective effect of income against CMC is larger for Whites than African Americans. This finding is a replication of the Blacks’ diminished return for the association between income and CMC.

We showed diminished return from income for African Americans. Similar to our findings, smaller health effects are shown for education and employment [24,25,28]. A recent study showed that the impact of employment on life expectancy in the U.S. is dependent on race, with smaller life expectancy gains following employment for African Americans compared to Whites. The study showed that while the effects were very large for White men with high education, there was no life expectancy gain for Black men [28]. Another study showed that education has a stronger effect on health behaviors [25] and life expectancy [24] of Whites compared to African Americans. Other similar findings are shown for affect, anger management, sleep quality, self-rated health, self-efficacy, and perceived control over life [42,43,44,45,46,47,48,49,50,51,52,53].

Due to the labor market preferences and practices, Whites enter better occupations that African Americans, which result in higher earnings and lower stress [54,55,56]. As a result, how these SES indicators such as education and employment result in health gains may vary by race [54,57,58,59].

The heterogeneity in the magnitude of the health return associated with SES indicators such as income, education, and employment, may be due to the structural racism embedded in American society. We argue that American society is designed and functions in a way that maximizes the benefits of Whites, and White men, and may even minimize the health return for African Americans, and African American men. Our argument does not support the premise that African Americans mismanage their resources, or that Whites know how to use resources more wisely. Rather, we argue that the U.S. social structure fails African Americans, even those able to climb the social ladder and become wealthy. Regardless of education attainment, wealth, or occupation, Blacks’ have higher physical health costs for social gains. The same is true for mental health, as high SES may be positively associated with depression and suicide among African Americans [23,60].

In addition, this study contributes to the theoretical models that explain health disparities in two ways. First, it provides support for Blacks’ diminished return, suggesting that socioeconomic factors consistently result in reduced health gains for African Americans compared to Whites [18]. Similar to this finding, a smaller effect of education on life expectancy is documented for African Americans than for Whites [24]. This theory introduces differential effects of the same resources as a mechanism for health disparities, and suggests that not all disparities are due to differential exposures between social groups [26,27]. Second, it shows that health of Whites, but not Blacks, declines more in the absence of SES indicators, such as income. This is in contrast to the Double Jeopardy [61], Triple Jeopardy [62], and Multiple Jeopardy [63] or Multiple Disadvantage [64] hypotheses. Most of these frameworks have traditionally conceptualized minority populations as vulnerable groups, suggesting that their health is more susceptible to the effects of any additional risk factor [63]. 

Other studies have also shown that race or the intersection of race and gender may alter the effects of SES indicators on other health outcomes, such as mortality. Hayward et al., for example, showed that an association of education on mortality was found for Whites but not African Americans [65]. Backlund et al. showed that for Whites with a high school diploma, each additional year of schooling had a stronger effect in reducing mortality. Among African Americans, however, there was a step reduction in mortality at 12 and 16 years of education, with constant slopes across the steps [66]. Everett et al. [67] and Cuttler [68] also suggested that there might be racial differences in the effects of education on health. Holmes and Zajacova showed that education is not “the great equalizer” of the health of Whites and African Americans [69]. 

Our findings on Blacks’ diminished return for the number of CMC is important, as previous research has shown that the number of CMC fully mediates the effect of race and SES on life expectancy [70] and self-rated health [71] in the U.S. That is, if policy-makers and public health officials eliminate the racial gap in the number of CMC, they should be successful in eliminating the resultant health disparities [72]. Our findings suggest that policies and programs should also aim to reduce Blacks’ diminished return as a strategy to eliminate health disparities [26,27]. 

In line with previous research [23,25,65,66], our findings agree with the argument by Williams that SES does not explain the effects of race on health [70]. In this view, increasing class does not promote the health of African Americans as it does for Whites, as race limits how individuals benefit from these SES resources [26,27]. This is also in line with the findings of LaVeist, who showed that Black-White disparities are very large even when African Americans earn more than USD170,000 annually [72].

Racism in the labor market and segregation may also be involved. To generate the same income, African Americans should have higher education compared to Whites [33,34,73]. Furthermore, racial inequity in pay, particularly in high-paying occupations, may result in a large racial gap between how high SES protects Whites and African Americans against poor health [72,73].

### 4.1. Implications

Our findings have policy implications. The diminished health return from SES resources is a major contributor of racial health disparities in the U.S. Policies that universally enhance SES indicators in the U.S. may not reduce, but may even widen these health disparities. To eliminate the persisting racial health disparities in the U.S., it is not enough to equalize the access to resources across populations; there is a need to develop policies that ensure that all social groups can benefit from such resources, regardless of their race. These findings advocate for additional investment in welfare as well as affirmative action in the U.S.

Our study also contributes to health disparities research methodology. Similar to previous work [23,25,65,66], there are non-linear, interactive, and multiplicative effects between race, gender, class, and place on health. Risk and protective factors interact with sociodemographic characteristics in their effects on health [74]. Previous researchers have advocated for future research to systematically test all potential interactions between race, ethnicity, and SES on health [39,74,75]. Disparities seem to be larger at the highest levels of SES, suggesting that the relative diminished return is greater at higher SES levels [18]. So, assuming the effects of the same risk and protective factors are similar across social groups is over-simplistic, and may result in misspecification of models that control for SES indicators in the pooled sample. 

### 4.2. Limitations

Our study is not free of limitations. The cross-sectional design of the study prevents us from establishing causal associations between income and CMC. Reverse causality between CMC and income cannot be ruled out. The White sample in the NSAL lives in proximity to African Americans, therefore, the results among Whites should be interpreted more cautiously. The study was limited in not measuring a wide range of confounders such as health care use and mental health. For instance, this study did not include contextual measures such as urbanity, place of residence, and neighborhood measures. In addition, this study did not collect data on spouse or partner SES, which may confound our findings. Any study that compares racial groups for the effects of the same risk factor on the same outcome may be impacted by the differential validity of the constructs across racial/ethnic groups. For instance, self-reported income or CMC may have higher validity among one race than another. In addition, type of medical condition was not considered in the study. Future research is needed to understand if this finding holds in other settings, cohorts, and age groups.

## 5. Conclusions

To conclude, race mitigates the health gain that follows income in the U.S. The effect of racial categorization is so pervasive that African Americans consistently gain less from the same SES indicators. Policies that universally increase the access of SES indicators to populations and ignore the fact that minority populations are always less able than the majority group to take advantage of the resources that become available to them may not be enough for the elimination of racial health disparities in the United States. In addition to policies that increase population access to education, jobs, and income, policies and programs should make efforts to equalize benefits that follow such resources. Such an aim can only be accomplished once the structural racism embedded in U.S. institutions is reduced. 

## Figures and Tables

**Table 1:** Descriptive statistics in the pooled sample and by race.

Characteristics	All		Whites		African Americans	
	%	95% CI	%	95% CI	%	95% CI
Gender						
Men	45.72	43.49–47.98	47.33	43.01–51.68	44.02	42.34–45.71
Women	54.28	52.02–56.51	52.67	48.32–56.99	55.98	54.29–57.66
Education (≥12 years) * ^a^						
Low	19.53	16.89–22.47	15.17	10.82–20.86	24.16	21.80–26.68
High	80.47	77.53–83.11	84.83	79.14–89.18	75.84	73.32–78.20
Married * ^a^						
No	51.94	48.02–55.84	45.91	38.50–53.51	58.35	56.24–60.42
Yes	48.06	44.16–51.98	54.09	46.49–61.50	41.65	39.58–43.76
	**Mean**	**95% CI**	**Mean**	**95% CI**	**Mean**	**95% CI**
Age (years)	43.47	42.01–44.93	44.82	41.95–47.70	42.04	40.96–43.13
Income (USD10,000) * ^b^	4.17	3.73–4.60	4.68	3.85–5.51	3.62	3.35–3.90
CMC	1.29	1.18–1.40	1.32	1.09–1.54	1.26	1.21–1.31

* *p* < 0.05 for comparisons of whites and African Americans. ^a^ Pearson Chi square, ^b^ independent samples *t* test. CMC: Chronic Medical Conditions. Source: National Survey of American Life (NSAL).

**Table 2:** Pearson correlations between study variables in the pooled sample and by race.

Characteristics	1	2	3	4	5	6	7
**All**							
1 Race (African Americans)	1.00						
2 Gender (Women)	0.05	1.00					
3 Age	−0.11 *	−0.01	1.00				
4 Education (≥12 years)	−0.09	−0.02	−0.19 *	1.00			
5 Married	−0.12 *	−0.16 *	0.01	0.07	1.00		
6 Income (USD10,000)	−0.15 *	−0.16 *	−0.02	0.28 *	0.38 *	1.00	
7 Chronic Medical Conditions (CMC)	−0.04	0.08	0.48 *	−0.20	−0.05	−0.15 *	1.00
**Whites**							
2 Gender (Women)	-	1.00					
3 Age	-	0.04	1.00				
4 Education (≥12 years)	-	0.00	−0.15 *	1.00			
5 Married	-	−0.16 *	−0.09	0.01	1.00		
6 Income (USD10,000)	-	−0.14 *	−0.09	0.21 *	0.39 *	1.00	
7 Chronic Medical Conditions (CMC)	-	0.09	0.47 *	−0.18 *	−0.10	−0.21 *	1.00
**African Americans**							
2 Gender (Women)	-	1.00					
3 Age	-	−0.02	1.00				
4 Education (≥12 years)	-	−0.02	−0.21 *	1.00			
5 Married	-	−0.15 *	0.02	0.07	1.00		
6 Income (USD10,000)	-	−0.16 *	−0.02	0.29 *	0.36 *	1.00	
7 Chronic Medical Conditions (CMC)	-	0.08	0.49 *	−0.21 *	−0.05	−0.14 *	1.00

* *p* < 0.05; Source: National Survey of American Life (NSAL).

**Table 3:** Summary of linear regressions between income and the number of chronic medical conditions in the pooled sample.

Characteristics	Model 1 Main Effects		Model 2 Model 1 + Interactions	
	B	95% CI	B	95% CI
Race (African Americans)	−0.03	−0.16–0.11	−0.14	−0.34–0.06
Gender (Women)	0.18 **	0.06–0.31	0.18 **	0.06–0.31
Age (Years)	0.04 ***	0.04–0.05	0.04 ***	0.04–0.05
Education (≥12 years)	−0.33 ***	−0.52–0.15	−0.34 ***	−0.53–0.16
Married	0.02	−0.10–0.13	0.02	−0.10–0.13
Income (USD10,000)	−0.06 ***	−0.08–0.04	−0.07 ***	−0.09–0.05
Income (USD10,000) * Race	-	-	0.03 *	0.00–0.06
Intercept	−0.43 *	−0.76–0.10	−0.37 *	−0.73–0.01

Outcome: Chronic Medical Conditions (CMC); * *p* < 0.05, ** *p* < 0.01, *** *p* < 0.001. Source: National Survey of American Life (NSAL).

**Table 4:** Summary of linear regressions between income and the number of chronic medical conditions in Whites and African Americans.

Characteristics	Model 1 Whites		Model 2 African Americans	
	B	95% CI	B	95% CI
Gender (Women)	0.12	−0.11–0.35	0.25 ***	0.18–0.33
Age (years)	0.04 ***	0.04–0.05	0.05 ***	0.04–0.05
Education (≥12 years)	−0.36 #	−0.77–0.05	−0.33 ***	−0.46–0.19
Married	0.08	−0.12–0.28	−0.05	−0.16–0.07
Income (USD10,000)	−0.07 ***	−0.10–0.04	−0.04 **	−0.06–0.01
Intercept	−0.24	−0.83–0.34	−0.64 ***	−0.83–0.46

Outcome: Chronic Medical Conditions (CMC); # *p* < 0.1, ** *p* < 0.01, *** *p* < 0.001, Source: National Survey of American Life (NSAL).
